# Optimising the breast radiotherapy planning pathway: a quality improvement project at a regional cancer centre

**DOI:** 10.1136/bmjoq-2025-004056

**Published:** 2026-07-27

**Authors:** Douglas Etheridge, Ryan David Lewis, Adam Henry Aitkenhead, Christopher Scott Rose, Nathan Proudlove

**Affiliations:** 1 Radiotherapy Physics, South West Wales Cancer Centre, Swansea Bay University Health Board, Swansea, Wales, UK; 2 The University of Manchester, Manchester, UK; 3 Christie Medical Physics and Engineering, The Christie NHS Foundation Trust, Manchester Academic Health Science Centre (MAHSC), Manchester, UK; 4 Division of Cancer Sciences, The University of Manchester, Manchester, UK; 5 Alliance Manchester Business School, The University of Manchester, Manchester, UK

**Keywords:** Radiotherapy, Quality improvement, Performance measures, Time-to-Treatment, Healthcare quality improvement

## Abstract

At the South West Wales Cancer Centre, breast radiotherapy accounts for 24% of the radiotherapy workload. In 2021, only 16% of patients started treatment within 14 days compared with the Welsh time-to-radiotherapy quality metric target of 80%. Delays risk poorer outcomes for patients and create inefficiencies as tasks are often batched and staff alternate between idle and peak workload periods.

Earlier analysis showed that only 1%–2% of the overall breast radiotherapy pathway time was value-adding activity. Key delays occurred at several stages of the pathway, where the total duration of each stage, including time spent waiting in the queue and time actively worked on, averaged around 50% longer than scheduled. For example, CT simulation was typically scheduled for 2 days but took more than 4 days while organ at risk (OAR) delineation was scheduled for 2 days but took approximately 2.5 days. Our improvement strategy targeted these delays through: (1) expansion of the technologist plan approval (TPA) pathway, (2) introduction of daily workflow meetings and clearer, standardised planning protocols and (3) implementation of artificial intelligence (AI) auto-contouring for OAR delineation. These changes were tested using the Model for Improvement, with statistical process control monitoring of metrics plus staff feedback.

The mean total radiotherapy breast pathway time from consent-to-treatment decreased from 19.5 days to 14.8 days, with patients commencing treatment within 14 days increasing from 16% to 55%. Use of the TPA pathway increased from 61% to 95% and plan queries reduced from 11% to 3%, reducing oncologist workload. Daily workflow meetings and improved protocols reduced average planning times from 4 days to 1.5 days and were valued by staff for improving workload equity and team cohesion. AI auto-contouring reduced OAR delineation from 2.3 to 0.6 days, though ongoing validation of contour quality is required.

**WHAT IS ALREADY KNOWN ON THIS TOPIC**

- Delays in the radiotherapy planning pathway are common, yet structured quality improvement methods have rarely been applied or evaluated, especially in the high-volume breast pathway.

**WHAT THIS STUDY ADDS**

- Targeted quality improvement interventions that streamline the pathway, through advanced practice roles such as technologist plan approval, daily workflow meetings and automation via artificial intelligence auto-contouring, produced sustained reductions in planning time, variation and oncologist workload without additional staffing and strengthened service resilience to improve timely access to treatment.

**HOW THIS STUDY MIGHT AFFECT RESEARCH, PRACTICE OR POLICY**

- The approach provides a transferable model for improving efficiency and meeting time-to-treatment standards across radiotherapy pathways and centres.

## Problem

The South West Wales Cancer Centre (SWWCC) is a regional cancer centre serving a catchment population of approximately a million people and treating around 2600 radiotherapy patients per year. Breast cancer is the most common cancer among women in the UK, with approximately 56 800 new diagnoses annually.^1^ Breast radiotherapy accounts for around 24% of all planned radiotherapy treatments at SWWCC, making it the largest single site of activity. The FAST-Forward trial demonstrated the safety and efficacy of reducing breast radiotherapy from 15 to 5 fractions, and this shorter schedule is now widely adopted.^2^ However, although this change in 2020 reduced the total number of treatment fractions and eased pressure on treatment capacity, breast radiotherapy remains a key determinant of departmental demand for CT simulation and treatment planning workload. This quality improvement project (QIP) formed part of a wider departmental programme of radiotherapy quality improvement (QI) focused on reducing pathway variation and improving workflow efficiency across all tumour sites. The breast radiotherapy pathway (BRTP) was selected for the first QIP because it represents the largest single planning workload, has well-established delegated roles such as technologist plan approval (TPA) and uses highly standardised protocols, making it an ideal model for testing and refining improvement methods before wider roll-out.

In 2019, local analysis of the BRTP showed that value-added activity accounted for only 1%–2% of the total pathway time,^3^ with the majority of time lost to waste delays and non-value-adding time (in lean terms: non-value-adding and ‘necessary non-value-adding’ steps).^4^ Key bottlenecks were identified at CT simulation, organ-at-risk (OAR) delineation, treatment planning and plan approval. The total duration of these stages, including both waiting and active work time, averaged around 50% longer than scheduled. For example, CT simulation was scheduled for 2 days but took more than 4 days, while OAR delineation was scheduled for 2 days but took about 2.5 days. These delays reflected operational inefficiency from uneven workflow rather than reduced individual staff performance. Tasks progressed in batches, producing alternating periods of low and high activity, a recognised form of system waste in lean process analysis. As a result, the department frequently breached the Welsh time-to-radiotherapy quality metric (WTRQM), which specifies that 80% of scheduled breast radiotherapy should commence within 14 days of patient consent and 100% within 21 days.^5^ Baseline data from 2021 confirmed the persistence of these issues, with a mean consent-to-treatment time of 19.5 days, only 16% of patients starting treatment within 14 days and 73% within 21 days.

Delays in initiating radiotherapy risk poorer oncological outcomes^6^ and cause significant stress for patients and staff.^7^ Workflow analysis indicated that these delays disrupted departmental efficiency by creating peaks of activity following periods of inactivity, particularly when oncologists reviewed multiple cases simultaneously. Staff surveys and informal feedback highlighted frustration with predictable plan queries, uneven workload distribution and the time burden of manual OAR delineation. These recurrent queries were better reflected by the overall plan query rate, which captured their frequency and cumulative impact on workflow.

### SMART aim

Our aim was to improve the efficiency of the BRTP with three goals (see the driver diagram,^8 9^ in online supplemental figure S1):

10.1136/bmjoq-2025-004056.supp1 Supplementary data

1. Improve performance against the 14-day and 21-day WTRQM cancer-wait targets.
2. Optimise the use of departmental resources.
3. Improve staff and patient satisfaction.

This QIP was initiated to achieve these aims. CT and treatment capacity constraints were recognised but were outside the scope of this project, as these would require capital investment and service-wide optimisation. Goal 1 provided a specific, measurable, achievable, relevant and time-bound (SMART) objective: that the planning pathway from vSim to check stage of the pathway should take no more than 10 days for at least 80% of patients by January 2024.^10^ (The ‘check stage’ refers to the final safety verification of the treatment plan, including review of plan quality and treatment parameters as defined within national guidance).^11^

The Model for Improvement (MFI) was used to guide system analysis,^4 8^ generate change ideas and test them through iterative plan-do-study-act (PDSA) cycles. This approach has been applied successfully in QIPs across hospital clinical sciences, including radiotherapy physics,^12^ medical physics,^13^ physiological sciences^14–16^ and life sciences.^17–19^

## Background

Timely access to radiotherapy is critical to optimise cancer outcomes. Delays between diagnosis and treatment increase the risk of tumour progression, reduce survival, negatively affect patient experience and impact capacity.^6 20 21^ Radiotherapy planning is particularly vulnerable to inefficiencies due to the number of tasks, staff groups and technologies involved.^22^

**Figure 1:**
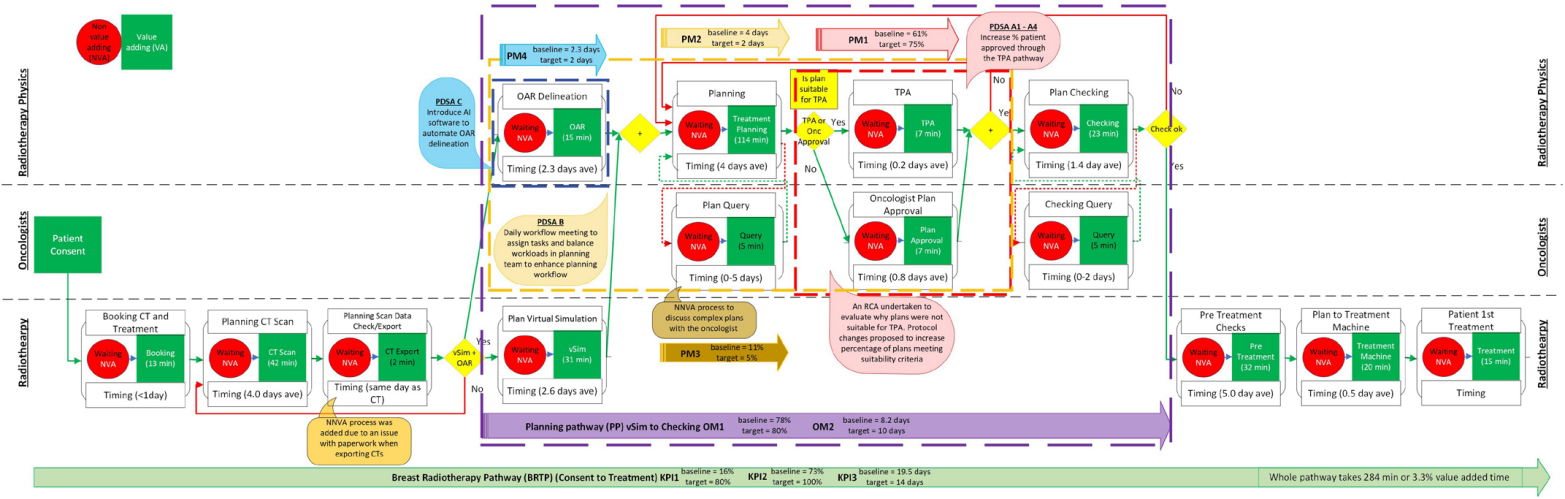
Process map of the whole breast radiotherapy pathway, showing the change ideas and the metrics of the QIP. IM, input metric; KPI, key performance indicator; OAR, organ at risk; OM, outcome metric; PDSA, plan-do-study-act; PM, process metric; QIP, quality improvement project; TPA, technologist plan approval; VA, value-adding; NVA, non-value-adding; NNVA, necessary non-value-adding; RCA, root cause analysis.

The BRTP is among the most complex pathways, involving CT simulation, virtual simulation (vSim), OAR delineation, treatment planning and plan approval (figure 1). Breast radiotherapy presents unique challenges due to techniques such as deep inspiration breath hold, introduced following the HeartSpare trial.^23^ As demand for breast radiotherapy continues to increase,^1^ these inefficiencies risk worsening compliance with the WTRQM.

QI approaches have streamlined pathways in other radiotherapy settings, such as electron radiotherapy treatment planning pathways,^12^ but there is limited published evidence applying structured QI methods to the BRTP. In 2019, a pathway-mapping workshop at SWWCC found that only 1%–2% of pathway time was value-adding activity, and reduced mean breast pathway times from 31 to 22 days, although inefficiencies persisted, particularly in treatment planning, plan approval and OAR delineation.^3^ These findings align with international evidence that radiotherapy pathways frequently experience delays and unnecessary variation.^24^

Emerging technologies and advanced practice roles offer opportunities for further improvement. The TPA pathway, in which plan approval is delegated from oncologists to radiotherapy physics staff, has been implemented across multiple radiotherapy pathways at SWWCC, including prostate, head and neck, gynaecology, colorectal, oesophageal and breast. This has reduced oncologist workload across these tumour sites. Daily workflow meetings are widely used in other healthcare settings and have been shown to improve task allocation and reduce bottlenecks. Artificial intelligence (AI)-based OAR auto-contouring is also increasingly recognised as a safe and effective method to reduce delineation times, including in National Institute for Health and Care Excellence(NICE) guidance.^25^ However, the impact of these interventions has not been previously evaluated across the entire BRTP.

## Measurement

The BRTP is complex, involving multiple handovers between radiographers, radiotherapy physics technologists (often referred to as dosimetrists in the UK and responsible for OAR delineation and treatment planning), clinical scientists and oncologists (see the process map in figure 1). Administrative staff and nurses also contribute at key points. This QIP focused on the work undertaken by the radiotherapy physics team and oncologists, particularly the treatment planning and plan approval stages of the pathway.

Data are recorded in our oncology information system, MOSAIQ® (Elekta AB, Stockholm, Sweden), where each step of the planning pathway is recorded as a Quality Check List (QCL) entry. Each QCL captures the start and finish times, and the staff member responsible. QCL data capture the total process duration, including both value-adding and non-value-adding (waiting) periods, enabling analysis of complete pathway efficiency.

For this project, a customised data extract was produced to obtain patient-level records for all breast cases since 2021. These were consolidated into a single dataset containing task start and finish times, duration and the staff member assigned. Reported times represent the total elapsed duration of each stage, including value-adding activity and non-value-adding waiting time, and any pauses in work. These components could not be separated, so it was not possible to determine which part of each pathway stage was most affected by the interventions. The final dataset was prepared and analysed using statistical process control (SPC) charts,^26 27^ based on the NHS SPC templates^28^ to detect shifts in performance over time.

At SWWCC, a radiotherapy physics dashboard (RTPDash) was developed for routine performance monitoring. It extracts QCL data from MOSAIQ® to provide an overview of performance across radiotherapy planning pathways, displaying indicators such as patient numbers by tumour site, stage and pathway durations as well as plan allocation by staff group. The RTPDash supports the monitoring of performance indicators, the early identification of bottlenecks and workload management. It complemented the QIP-specific dataset by enabling monitoring of other pathways to assess whether changes in the breast pathway influenced wider service performance.

Root cause analysis^4 8^ identified several interrelated factors contributing to delays. Restrictive and underspecified protocols limited staff autonomy and failed to provide sufficient guidance for consistent, informed decision-making. Narrow TPA criteria necessitated that many plans be reviewed by oncologists. In most cases, the oncologist’s response was predictable, indicating that revised protocols could safely reduce unnecessary reviews and workflow interruptions. Workflow variation and inefficiencies also contributed, with inconsistent task allocation, batching of work and multiple handovers, creating variability and avoidable delays. Finally, manual tasks, such as OAR delineation, were time-consuming and further prolonged the overall pathway.

Baseline measurement used data from January to September 2021 to characterise the BRTP and identify targets for improvement. Data prior to January 2021 were excluded because the COVID-19 pandemic disrupted patient referrals and workflow across radiotherapy pathways. The baseline dataset, therefore, represents a 9-month period encompassing 188 breast patients. Analysis confirmed that value-adding activity accounted for very little (around 3.3%) of the total pathway time, with delays occurring at several stages of the process.

**Figure 2:**
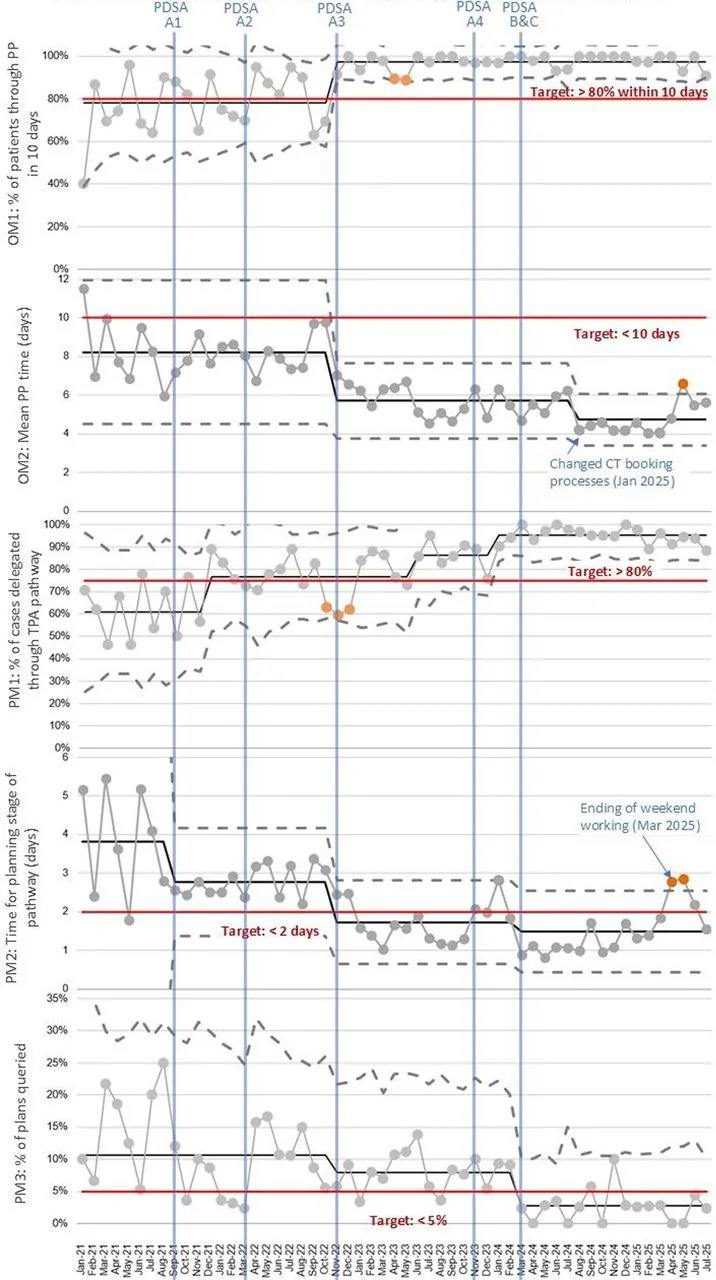
Stacked statistical process control (SPC) charts of key performance metrics. OM1 (monthly percentage of patients through the PP in 10 days); OM2 (mean planning pathway time in days); PM1 (monthly percentage of cases delegated through the TPA pathway); PM2 (monthly mean for the planning stage of the pathway in days); PM3 (monthly percentage of the plans that have a query). Coloured data points are periods of unusually good or poor performance. PP, planning pathway; OM, outcome metric; PDSA, plan-do-study-act; PM, process metric; TPA, technologist plan approval.

To assess improvement, the WTRQM cancer wait targets were used as higher-level key performance indicators, and outcome and process metrics were defined for the pathway stages targeted by this QIP. All metrics were calculated monthly. Figure 1 illustrates the location of these metrics within the broader pathway, and figure 2 and online supplemental figure S2 display the baseline performance.

10.1136/bmjoq-2025-004056.supp2 Supplementary data

- Key performance indicators (KPI)
  - KPI1: percentage of patients starting treatment within 14 days of consent. Baseline 16%, WTRQM target 80%.
  - KPI2: percentage of patients starting treatment within 21 days of consent. Baseline 73%, WTRQM target 100%.
  - KPI3: mean time from consent to start of treatment. Baseline 19.5 days, target 14 days.
- QIP outcome metrics (OM)
  - OM1: percentage of patients through the planning pathway (from vSim to check) within 10 days. Baseline 78%, target >80% (consistently).
  - OM2: mean time through the planning pathway (vSim to check). Baseline 8.2 days, target <10 days.
- Process metrics (PM) selected to measure targeted drivers
  - PM1: proportion of plans approved via TPA. Baseline 61%, target >75%.
  - PM2: breast planning time. Baseline 4 days, target <2 days.
  - PM3: proportion of plans queried. Baseline 11%, target <5%.
  - PM4: OAR delineation time. Baseline 2.3 days, target <2 days.
- Input metric (IM)
  - Workload: total number of patients through the pathway each month. Baseline 22.

## Design

The QIP team comprised radiotherapy physics clinical scientists, technologists and clinical oncologists, the staff groups directly responsible for processes identified as bottlenecks in the baseline analysis: OAR delineation, treatment planning and plan approval. Although not the full multidisciplinary team, these members held the operational knowledge and authority required to design and test pathway changes.

Root cause analysis identified three contributory issues: restrictive protocols and narrow TPA criteria, which created unnecessary oncologist reviews; inconsistent task allocation and batching, which prolonged planning times; and manual OAR delineation, which added substantial necessary non-value-adding time. These findings informed three linked change concepts that formed the interventions for this QIP: expansion of the TPA pathway (concept A), introduction of daily workflow meetings (concept B) and implementation of AI OAR auto-contouring (concept C); see figure 1, online supplemental figure S1 and table 1.

**Table 1:** Summary of PDSA cycles

Cycle	Plan/prediction	Do	Study (results)	Act/conclusion
Change idea A: improvement in the TPA pathway and scope of practice: four PDSA cycles				
**A1** September 2021–March 2022	Expand TPA scope to reduce oncologist queries	Implement shielding protocol for lung/heart exceeding tolerance without oncologist review	**KPI3:** no change (19.5 days) **OM2**: no change (8.2 days) **PM1:** TPA: **↑** 61% → 75% **PM2:** planning time: **↓** 4.0 → 2.8 days **PM3:** plan queries: no change (11%)	Retain change. Explore further expansion of prescriptions.
**A2** March 2022–November 2022	Test additional prescriptions in TPA scope	Expanded TPA criteria to include more prescriptions	**KPI3:** no change (19.5 days) **OM2**: no change (8.2 days) **PM1:** TPA: no change (75%) **PM2:** planning time: no change (2.8 days) **PM3:** plan queries: no change (11%)	Minimal impact; retain change anyway. Further refine approval criteria in A3.
**A3** November 2022–November 2023	Further expand TPA for complex breast cases	Extend TPA to include large breast cases with anterior boost; streamlined shielding criteria	**KPI3:** reduced **↓** 19.5 → 14.8 days **OM2**: reduced **↓** 8.2 → 5.7 days **PM1:** TPA: **↑** 75% → 86% **PM2:** planning time: **↓** 2.8 → 1.7 days **PM3:** plan queries: **↓** 11% → 8%	Adopt. Incorporate into protocol.
**A4** November 2023	Include previously resolved oncologist queries in TPA	Allow plans with documented oncologist-approved queries to proceed via TPA without further review; reiterate pathway to technologists	**KPI3:** remained 14.8 days **OM2**: remained 5.7 days **PM1:** TPA **↑** 86% → 95% **PM2:** planning time: **↓** 1.7 →1.5 days **PM3:** plan queries: **↓** 8% → 3%	Adopt. Incorporate into protocol.
Change idea B: introduction of daily workflow meetings: 1 PDSA cycle				
**B** February 2024	Introduce daily workflow meetings to improve task allocation and reduce batching	Implement 10 min daily meetings at 10:00, chaired by rotating staff on a 2-week cycle	**KPI3:** remained 14.8 days **OM2**: reduced **↓** 5.7 → 4.7 days **PM2:** planning time: remained (1.5 days) Reduced unassigned tasks. Staff feedback highlighted clearer responsibilities and stronger team cohesion	Embed as routine practice. Continue to monitor workload distribution.
Change idea C: introduction of AI auto-contouring: 1 PDSA cycle				
**C** February 2024	Use AI auto-contouring to reduce OAR delineation time	Pilot AI auto-contouring for heart and lung, with outputs reviewed and edited by technologists	**KPI3:** remained 14.8 days **OM2**: reduced **↓** 5.7 → 4.7 days **PM4:** OAR delineation **↓** 2.3 → 0.6 days Staff reported improved efficiency but emphasised need for validation and training	Retain change. Further audit required to confirm reduction in value-added time and validate clinical acceptability of AI contours.

Metrics: KPI3: time from consent to start of treatment, OM2: time for vSim to check, PM1: proportion of breast plans approved by TPA, PM2: breast planning time, PM3: plan query rate, PM4: OAR delineation time; green arrows emphasise positive changes.

AI, artificial intelligence; KPI, key performance indicator; OAR, organ at risk; OM, outcome metric; PDSA, plan-do-study-act; PM, process metric; TPA, technologist plan approval; vSim, virtual simulation.

These interventions were expected to be effective because each targeted a specific pathway issue. Expanded TPA criteria were expected to reduce predictable queries and associated workflow interruptions. Daily workflow meetings aimed to smooth workload variation by clarifying task ownership and prioritising urgent cases. AI auto-contouring was expected to reduce delineation time and variation by eliminating a manual component of the task.

Team members were engaged through regular planning meetings, informal discussions and iterative review of protocol drafts. Oncologist engagement was particularly important for TPA expansion; their feedback shaped stepwise broadening of criteria and ensured continued clinical oversight. Technologists contributed to the design of workflow meetings and provided early testing of AI tools, informing decisions about training requirements and integration into existing software systems.

Potential challenges were identified at the design stage. These included ensuring oncologist confidence in the expanded TPA scope, embedding workflow meetings without adding administrative burden and validating AI contours to maintain clinical safety. To support sustainability, revisions to TPA criteria were incorporated into formal protocols and standard operating procedures. Workflow meetings were designed to be brief and structured, with chairing responsibilities rotated among staff. AI implementation was planned as a phased roll-out with continued audit, training and monitoring of contour quality.^25^

Together, these measures were intended to create a reliable, scalable and sustainable set of interventions that could be embedded into routine planning practice and extended to other tumour sites.

## Strategy

The MFI framework was applied through iterative PDSA cycles to test and refine the three change concepts, each targeting a specific stage of the pathway (online supplemental figure S1). Data were monitored continually using SPC charts and staff feedback to inform the adoption or refinement of changes (table 1, figure 2 and online supplemental figure S2).

### Change concept A: improvement in the TPA pathway and scope of practice (four PDSA cycles)

The aim of this change concept was to increase the percentage of plans suitable for the TPA pathway and reduce avoidable oncologist reviews. The prediction was that widening TPA criteria would reduce predictable queries, improve workflow continuity, maintain quality and safety and release oncologist capacity.

In cycle A1, shielding rules were amended to allow planners to add heart or lung shielding without oncologist consultation. TPA approvals (PM1) increased after a delay because the revised TPA criteria required incorporation into the protocol and were adopted once the updated guidance was in place. Planning time (PM2) decreased, and query rates (PM3) remained unchanged. This confirmed that some routine queries could be safely addressed through revised guidance.

In cycle A2, additional prescriptions were added to the TPA scope with minimal effect. In cycle A3, criteria were expanded to include large breast cases with anterior boost fields, and shielding rules were simplified. This produced clear improvements across all metrics and was sufficient to impact our QIP OMs and higher-level KPIs (see figure 2 and online supplemental figure S2).

Cycle A4 allowed plans with previously resolved oncologist queries to progress directly through TPA. This reduced query rates (PM3) further and increased approvals (PM1) to 95%. Although not all cycles produced measurable gains, each provided important information that supported the safe and incremental refinement of the protocol.

### Change concept B: introduction of daily workflow meetings (one PDSA cycle)

The aim of this intervention was to reduce batching and smooth task allocation. The prediction was that a short daily meeting would improve clarity of responsibilities and reduce delays. A 10 minute meeting was introduced at 10:00 with rotating chairing responsibilities. Planning time remained stable, but mean pathway time (OM2) improved after a time lag. Feedback confirmed better communication and clearer task ownership, although some workload variation persisted for staff with narrower scopes of practice. This confirmed the importance of ongoing monitoring and targeted training.

### Change concept C: introduction of AI auto-contouring (one PDSA cycle)

The aim of this change was to reduce the duration of the OAR delineation task. The prediction was that AI would reduce manual necessary non-value-adding work and reduce variation. A pilot of AI for heart and lung contours was introduced, and delineation time decreased from 2.3 to 0.6 days (PM4). Staff reported improved efficiency but noted the need for training and contour validation. Successful AI integration depends on familiarity with editing tools and confidence in contour quality. This feedback informed plans for further improvement, such as automatic initiation of procedures after CT acquisition and additional audits.

Together, the PDSA cycles demonstrated progressive improvement, clarifying which refinements were effective, which were neutral, and which required further development. The iterative nature of the strategy helped build confidence in the interventions and supported their adoption into routine practice.

## Results

The interventions produced sustained improvements across outcome, process and balancing measures. The driver diagram (online supplemental figure S1) illustrates the relationship between the interventions and measured outcomes, with the SPC analysis demonstrating clear and sustained improvement across the pathway following implementation of the change ideas (figure 2 and online supplemental figure S2). The proportion of patients completing the planning pathway within 10 days (OM1) increased from 78% to 97%, and the mean planning pathway time (OM2) reduced from 8.2 to 4.7 days. The SPC charts show the system is now statistically capable of meeting these internal targets.

Improvements on the WTRQM were also substantial. At baseline, the mean consent-to-treatment time (KPI3) was 19.5 days, and 16% of patients started treatment within 14 days (KPI1). By mid-2024, consent-to-treatment time had decreased to 14.8 days (KPI3), with 55% of patients starting within 14 days (KPI1) and 90% within 21 days (KPI2). However, further improvement is required to reach national targets and reduce variation.

The process measures show TPA approvals (PM1) increased from 61% to 95%, while query rates (PM3) decreased from 11% to 3%. Planning time (PM2) reduced from 4 days to 1.5 days, and OAR delineation time (PM4) reduced from 2.3 days to 0.6 days following AI implementation. These improvements were achieved despite unchanged staffing levels and an increasing workload since the baseline (see the IM graph, online supplemental figure S2), indicating that the interventions directly contributed to the observed gains. Changes to CT booking and vSim processes in August 2024 (outside the scope of this QIP) may have contributed to an improvement in OM2, although this has not led to further improvements in the WTRQM KPIs (online supplemental figure S2).

Contextual factors interacted with the interventions. Commissioning of the new Monaco treatment planning system (Elekta AB, Stockholm, Sweden) from January 2024 created additional workload and reduced planning capacity as staff adapted to new processes and migrated tumour-site pathways onto the platform. Breast planning has not yet been implemented in Monaco, but commissioning activity has placed pressure on the wider planning service. Data completeness was assessed by comparing extracted QCL records with the RTPDash. Missing data were minimal and did not influence the interpretation of results.

Staff turnover contributed to periods of greater variation, while the introduction of workflow meetings helped stabilise task allocation and communication during these phases. Workload distribution remained uneven in 2023 and 2024, with 1 technologist completing 35% of breast planning tasks despite 10 staff being trained. By early 2025, this improved to three planners each completing about 22% of cases. The ending of weekend working in March 2025 may have contributed to the recent increase in planning time, as indicated by the orange points on PM2 (figure 2).

There were no major unintended effects impacting patient safety. AI implementation required additional contour validation and training, but produced no unexpected failures.

Consultant oncologist time savings were achieved (see online supplemental table S1). Introduction of TPA in 2020 reduced oncologist approvals to 39%, releasing approximately 67 hours of consultant time annually. The current QIP reduced this further to 5%, releasing an additional 37 hours per year and saving approximately £1812. Although the financial savings were modest and do not include setup or administrative costs, the increased availability of oncologists for clinical and leadership work represents important service value.

Overall, the interventions produced sustained improvements in pathway performance, reduced variation and strengthened workflow stability compared with baseline. RTPDash monitoring showed that learning from the breast TPA work was applied to other tumour sites, increasing the use of their TPA pathways and producing similar efficiency gains. AI contouring was also introduced to other sites, producing comparable reductions in delineation time. These changes did not impact higher-level KPIs because CT and treatment machine capacity continued to limit how soon patients could start treatment.

## Lessons and limitations

This project demonstrated that meaningful improvement can be achieved in a complex radiotherapy planning pathway through structured QI methods. Several lessons emerged. First, expanding the TPA pathway through stepwise protocol changes was effective in reducing oncologist workload and improving planning efficiency. Incremental testing and oncologist oversight ensured changes maintained quality and safety. Second, small operational adjustments, such as daily workflow meetings, improved communication, workload visibility and team cohesion, stabilising task allocation during periods of staff turnover and operational pressure. Third, AI auto-contouring reduced non-value-added work, although successful adoption required training, contour validation and careful integration into existing workflows. Staff needed time to develop confidence in editing AI-generated structures and to recognise where manual adjustment remained essential.

Further optimisation of AI workflows is expected to deliver additional gains in efficiency and consistency. Planned developments include automatic initiation of AI immediately after CT simulation, triggered through DICOM header data, so that contours are available for review as soon as imaging is complete. This automation is likely to be most beneficial for tumour sites with a larger number of OARs and more complex delineation requirements. As AI becomes routine across tumour sites, its impact on value-added and non-value-added time will be evaluated in more detail.

There were several limitations. The project focused on a single radiotherapy pathway within a larger department and took place during a period of parallel service changes, including the commissioning of a new treatment planning system and staff turnover. These factors introduced variability, making it difficult to attribute all observed changes solely to the interventions. Although planning performance was monitored through the RTPDash, the data did not distinguish between scheduled and elective pathways, limiting analysis of variation caused by concurrent treatments. This primarily affects consent to CT and pretreatment stages, which were outside the scope of this project. Workload imbalance in 2023 and 2024, where one technologist completed a disproportionate share of breast plans, also highlighted the need for ongoing review of workforce resilience, although this improved in 2025. It is also difficult to isolate or attribute effects on the OM to a single change idea, due to overlap between interventions and their target outcomes.

For patients, delays within the BRTP can adversely affect experience, contributing to increased anxiety, uncertainty and reduced satisfaction with care, and have the potential to negatively impact both psychological well-being and treatment outcomes. However, since only workflow-based metrics were monitored through the RTPDash, it was not possible in this study to evaluate the impact of workflow improvement on the patient experience.

The specific staffing model, workforce skill mix and IT infrastructure within the centre limit generalisability. In particular, variation in training, roles and responsibilities between radiotherapy professionals across institutions may influence the feasibility and impact of similar interventions. However, the underlying principles of delegated roles, improved protocols, AI and active workflow management through regular meetings are transferable across centres. Local adaptation would be required, and results may differ in centres with different planning systems or governance frameworks.

Chance and confounding may also have influenced outcomes during periods of service pressure. However, the sustained improvement observed and the alignment between predicted and actual effects suggest that the interventions produced genuine process change.

The introduction of ultrahypofractionated regimens, such as those evaluated in the FAST-Forward trial, has reduced the number of treatment fractions delivered and increased linear accelerator capacity. However, this does not directly reduce workload within the BRTP as it may introduce additional complexity at certain stages. While this represents an important contextual factor influencing overall service capacity, its impact on the BRTP was not directly measured within the scope of this QIP.

If this work were repeated, earlier attention would be given to more equitable technologist workload allocation and to developing an automated audit for AI contour quality. A more detailed categorisation of pathway types would support finer analysis. Additional improvement work is also planned to optimise the booking-to-CT stage, which contributes significantly to pathway delays. Closer alignment between CT availability and consultant clinics, where patient consent is obtained, may reduce avoidable waiting and further improve compliance with WTRQM. Continued monitoring and iterative refinement will remain essential as these interventions are extended to other tumour sites.

## Conclusion

This QIP has shown that structured QI methods can deliver meaningful improvement within a complex radiotherapy planning pathway. The existing evidence highlights the importance of timely treatment for both patient outcomes and service capacity; nevertheless, delays in radiotherapy planning are well-documented. This work demonstrates that targeted operational changes, clearer pathways, updated protocols and appropriate use of technology can have a measurable and sustained impact on planning performance.

The project met its primary aims. Performance against WTRQM improved, variation reduced and planning efficiency increased without additional staffing. The measures used were appropriate for monitoring pathway performance, and the combination of outcome, process and workflow metrics provided a balanced view of change. The cost analysis showed modest financial savings, but a significant release of consultant oncologist time, representing a valuable benefit in the context of national workforce pressures.

Service sustainability is supported by the incorporation of revised TPA criteria into formal protocols, routine use of daily workflow meetings and the integration of AI auto-contouring into standard practice. Although external pressures influenced performance during the project period, the improvements have been sustained, suggesting that the changes are robust. Continued monitoring through the RTPDash will be essential to ensure that performance remains stable, particularly during commissioning of the new treatment planning system.

The approach could be replicated in other centres, although adaptation would be required to account for local workforce structures, planning systems and governance arrangements. Delegated advanced practice roles, structured communication processes and AI-supported delineation are widely transferable. Further development is planned to optimise the booking-to-CT stage, strengthen automated audit of AI contours and extend the interventions to other tumour sites.

Overall, the project has demonstrated that relatively small but well-targeted changes can improve pathway performance and staff experience and can support cancer centres in meeting national standards. Ongoing evaluation will help determine the broader impact on patient experience and ensure that the gains achieved continue to be built on.

## Data Availability

Data are available upon reasonable request. The datasets generated and/or analysed during the current study are not publicly available due to institutional governance and patient confidentiality considerations but are available from the corresponding author on reasonable request, subject to appropriate approvals.
