# Supplementary material for: Optimising the breast radiotherapy planning pathway: a quality improvement project at a regional cancer centre
Source: BMJ Open Qual. 2026 Jul 27;15(3):e004056. doi: 10.1136/bmjoq-2025-004056 (PMC13404411; doi:10.1136/bmjoq-2025-004056)
Supplement: Supplementary file 1 [file bmjoq-15-3-s001.pdf]

Figure S1: Driver Diagram

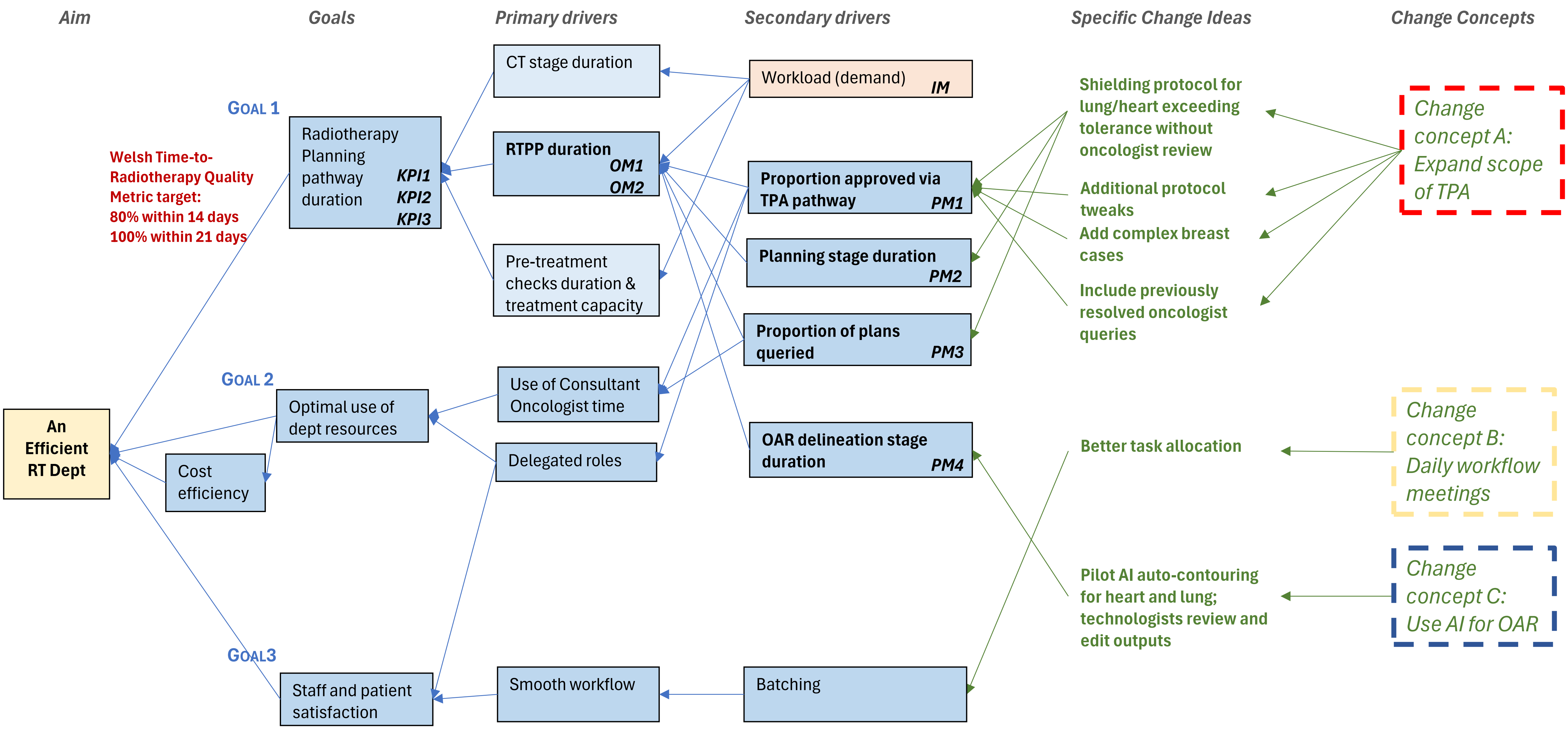

Table S1: Cost Model

Breast TPA cost analysis

	Salary Yearly	Salary Hourly
Consultant Clinical Oncologist (CCO)	£160,000.00	£ 82.05
Agenda for change Band 7 (Advanced Technoloist) (Tech)	£ 65,525.00	£ 33.60

		Approval Rate	Number Patients	Approval Time	Total Minutes	Total Hours	Hourly Cost CCO	Hourly Cost Tech	Cost Difference	Saving	
Pre 2020	Approval Rate	100%	440	15	6600	110.0	£9,025.64	£3,696.28	£5,329.36	£5,329.36	Saving assuming 100% of work transferd from CCO to Tech
2021	Tech	61%	268	15	4026	67.1		£2,254.73	£5,774.73	£1,811.98	Saving associated with this QIP assuming constant 440 patients per year
	CCO	39%	172	15	2574	42.9	£3,520.00				
2025	Tech	95%	418	15	6270	104.5		£3,511.47	£3,962.75		
	CCO	5%	22	15	330	5.5	£451.28				

Cost saving per patient									
Tech		1	15	15	0.3		£8.40	£28.91	£28.91
Onc		1	15	15	0.3	£20.51			
