# Supplementary figures and images for: Optimising the breast radiotherapy planning pathway: a quality improvement project at a regional cancer centre
Source: BMJ Open Qual. 2026 Jul 27;15(3):e004056. doi: 10.1136/bmjoq-2025-004056 (PMC13404411; doi:10.1136/bmjoq-2025-004056)

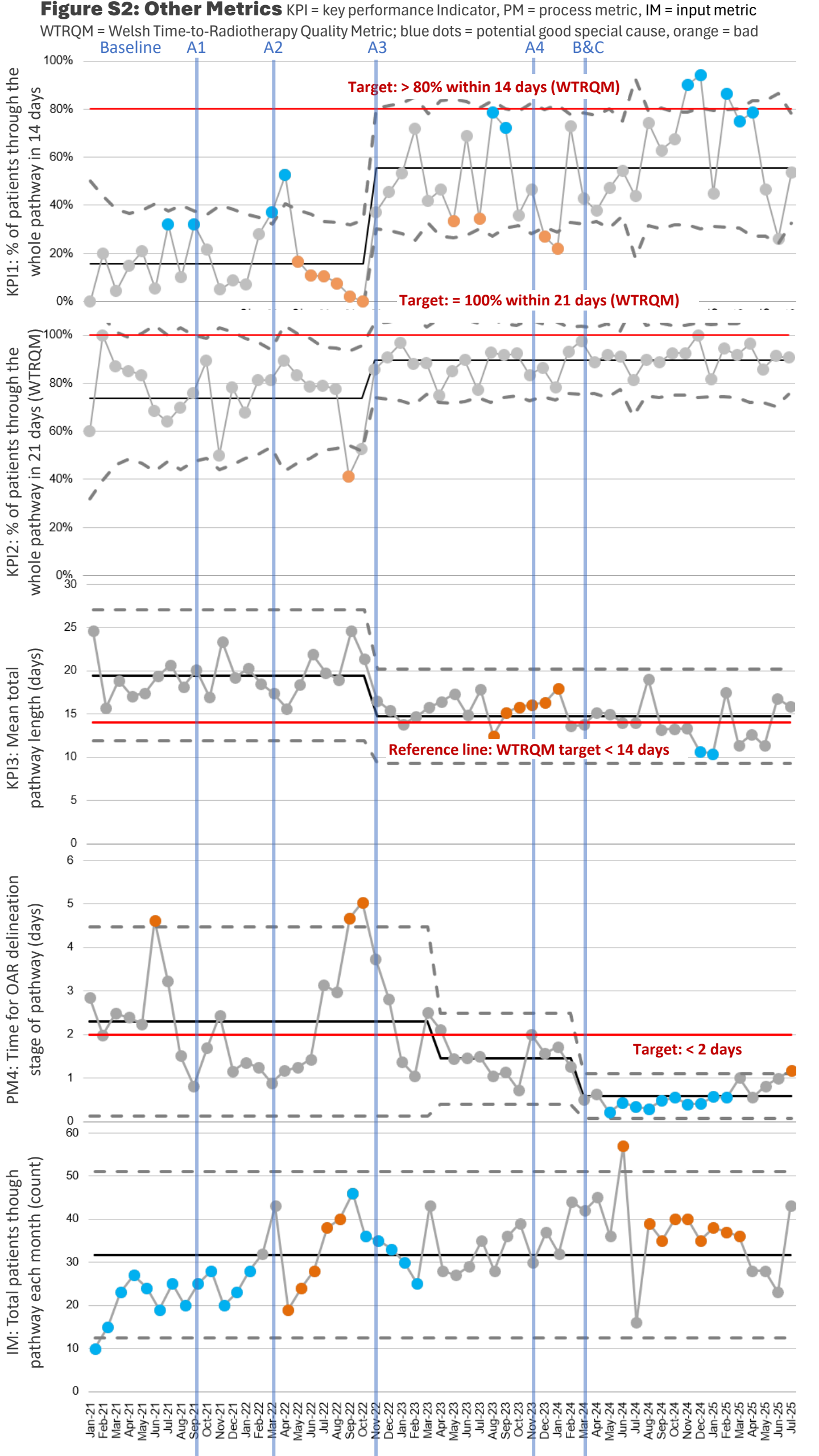

Supplement: Supplementary file 2 [file bmjoq-15-3-s002.pdf]
